# Retraction: Notch-1 Signaling Promotes the Malignant Features of Human Breast Cancer through NF-κB Activation

**DOI:** 10.1371/journal.pone.0283750

**Published:** 2023-03-23

**Authors:** The PLOS ONE Editors

Following the publication of this article [1], concerns were raised regarding results presented in Figures 2, 7, 9, and 10. Specifically,

- In Figure 2C there appear to be repetitive elements within and between the control panel and the scrambled shRNA panel.
- In Figure 7A, there appear to be repetitive elements within the following panels:
  - Vector 0h
  - Scrambled shRNA 0h
  - shRNA 0h
- The Figure 9B β-actin panels appear similar, despite being used to represent different samples (total protein, cytosolic extract, and nuclear extract).
- Figure 10, there appear to be repetitive elements within and between the lanes presented in this blot.

The authors provided underlying data and replacement images for the Figure 2C, 7A, and 9B results, but disagreed with the concerns raised with Figure 10, stating that there are differences between the lanes and that the level of similarity is normal and reasonable. The data provided for editorial review did not resolve the above concerns.

In light of the concerns affecting multiple figure panels that question the integrity of these data, the *PLOS ONE* Editors retract this article.

LL, TL, HY, CW, and YL did not agree with the retraction and stand by the article’s findings. FZ and JL either did not respond directly or could not be reached.

## References

[1] Li L, Zhao F, Lu J, Li T, Yang H, Wu C, et al. (2014) Notch-1 Signaling Promotes the Malignant Features of Human Breast Cancer through NF-κB Activation. PLoS ONE 9(4): e95912. doi: 10.1371/journal.pone.0095912 24760075 PMC3997497

